# Mandibular Unilateral Paresthesia Following Implant Placement and Clinical Management: Clinical Case Report

**DOI:** 10.7759/cureus.64001

**Published:** 2024-07-07

**Authors:** Shivakumar Baskaran, Hariharan Ramakrishnan, Deepavalli Arumuganainar

**Affiliations:** 1 Department of Periodontics, Ragas Dental College and Hospital, Chennai, IND; 2 Department of Prosthodontics and Implantology, Thai Moogambigai Dental College and Hospital, Dr MGR Educational and Research Institute, Chennai, IND; 3 Department of Periodontics, Saveetha Dental College and Hospitals, Saveetha Institute of Medical and Technical Sciences, Saveetha University, Chennai, IND

**Keywords:** implant retrieval, implant dentistry, dental implant complications, inferior alveolar nerve injury, mandibular paraesthesia

## Abstract

Dental implantology has been considered the mainstay in the rehabilitation of partial or complete edentulism. Nevertheless, complications and failures are occasionally encountered, and the most significant is the neurosensory disturbance. It not only causes persistent discomfort to the patient but frequently degrades the patient’s oral health-related quality of life, even leading to a negative psychological impact. This paper presents a case report of a 65-year-old male patient who underwent the replacement of his missing tooth in the right mandibular region (46) with an implant-supported prosthesis two years ago. Since then, he has been experiencing numbness in the right side of the lip and occasional drooling of saliva from the right corner of the mouth. Clinical examination revealed the presence of a prosthetic crown supported by an implant in relation to 46 with inflamed and enlarged gingiva in the region with paresthesia of the right lower lip region. Radiological examination with orthopantomogram (OPG) and cone beam computed tomography (CBCT) revealed that the implant apex rested on the inferior alveolar nerve canal. Careful surgical retrieval of the well-osseointegrated implant was performed under local anesthesia in about seven days. The patient gradually experienced neurosensory improvement, and the paraesthesia was completely resolved in a six-week period. After complete recovery, as evaluated with an objective and subjective assessment, the edentulous site was successfully restored with a provisional fixed partial denture.

## Introduction

Dental implantology has revolutionized dentistry in the replacement or rehabilitation of partially or completely edentulous sites. Due to the extensive use of dental implants in the past two decades, the associated complications and failures have also been proportionately increased. Several studies have suggested that one of the frequently encountered complications is injury to the inferior alveolar nerve, the lingual nerve, or the mental nerve [1]. The nerve injury associated with implant placement surgeries may occur either during the administration of local anesthesia, osteotomy site preparation, or during implant insertion [2]. The patients may experience mild to excruciating pain. In addition, the symptoms may be debilitating such that they affect one's routine activities like speech and mastication. The nerve injury may result in partial or complete loss of sensation, namely, the perception of touch, temperature, or pressure [2]. Such injuries may vary from mild, short-lived neurosensory disturbance to complete paresthesia and even dysfunction [3]. Studies on implant placement have suggested a range of 1.7 to 43.5% of patients reporting dysesthesia and 5-15% reporting complete paresthesia after one year [4]. It is demonstrated that the recovery from nerve injury is dictated by the initial severity of the injury [5].

A recent systematic review aimed to assess the complications of surgical inferior alveolar nerve (IAN) reposition techniques for implant-based rehabilitation in patients with severe mandibular atrophy in seven studies found a 99.26% implant survival rate, with persistent neurosensory problems being the most common complication [6]. A meta-analysis revealed that mandibular implant placement significantly increases the risk of neurosensory disturbance, with many patients experiencing spontaneous recovery. Clinicians should take precautions to prevent complications during implant placement, and more randomized controlled trials are needed to understand factors contributing to altered sensation [7].

The diagnosis of varying degrees of paresthesia depends on a thorough clinical examination considering the patient's comprehensive medical history. The patient's response to the thermal, electrical, and mechanical stimuli is key in confirming the diagnosis. Seddon et al. have delineated nerve injuries into three forms based on the severity of the injury, the prognosis, and the recovery time, namely neuropraxia, axonotmesis, and neurotmesis, with neuropraxia being the mildest form offering the best prognosis. In contrast, neurotmesis is the most severe form, presenting the worst prognosis [8]. The sensory disturbance arising from nerve injury results in an unpleasant experience for the patient and the clinician.

Thus, effective management of sensory disturbances is utmost warranted with either conservative or surgical treatment almost immediately after the diagnosis of injury has been made. Early diagnosis and prompt treatment result in better prognosis, as the peripheral nerve injuries are more likely to become permanent with increased duration between injury and treatment. Another potential negative impact of delayed diagnosis and treatment is an increase in the pressure on the peripheral nerves, leading to deranged neural microcirculation, ultimately leading to focal demyelination, which may progress to tunnel syndrome [9].

With the current availability of an extensive range of diagnostic and therapeutic methods, detecting and managing such injuries have become like any other routine task. By and large, a thorough basic examination and analysis of the underlying cause, the extent and the severity of the injury, or timely referral to a specialist are mandated to achieve the best possible treatment outcomes.

This case report describes a 65-year-old male patient in whom an endosseous implant placement in the right mandibular molar region led to sensory impairment that persisted for two years and was completely resolved with conservative surgical management.

## Case presentation

Case description and patient information

A 65-year-old male patient presented to the dental outpatient department with a primary complaint of numbness on the right side of the lower lip and mental region, along with occasional drooling from the right corner of the mouth for the past two years. He also reported an episodic burning sensation in the right inner cheek area. The patient indicated that these symptoms began approximately two days after undergoing implant placement surgery for a missing tooth in the right lower posterior region. He also complained of intermittent dull, throbbing pain in the right lower posterior tooth region. His medical history revealed that he is a known cardiac patient with a myocardial infarction that occurred 20 years ago, for which he was appropriately treated and is currently on medication. Upon general examination, his vital signs were within normal limits.

Clinical examination

**Figure 1:**
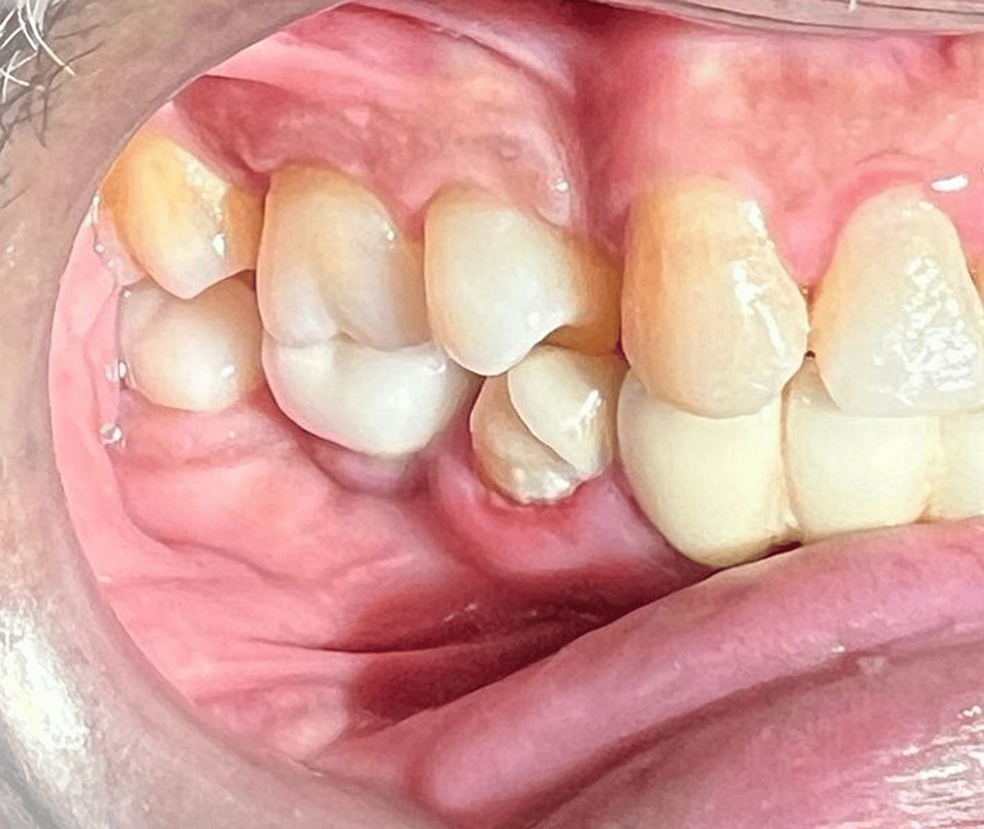
Clinical photograph of 46 with a prosthetic crown and 45 with clinical signs of inflammation

Clinical examination revealed the presence of a prosthetic crown supported by the implant in relation to 46. The gingiva was edematous, and there was bleeding on probing. The probing depth was recorded to be 5 mm. The crown, in relation to 46 and the implant, remained stable. The periodontium in relation to 45 showed clinical signs of inflammation, namely, redness and enlarged gingiva, and had been subjected to root canal therapy. No other abnormality was detected in the region of interest or extra orally. Soft tissue sensitivity was evaluated using a periodontal probe and a cotton pellet soaked in ethyl chloride. It demonstrated a complete loss of tactile and thermal sensation in the right lower lip and mental region, also involving the right side of the chin skin (Figure 1).

Radiological evaluation

**Figure 2:**
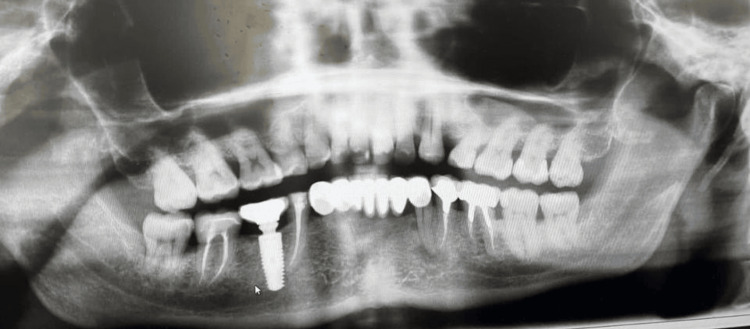
Orthopantanogram showing implant apex impinging on the inferior alveolar nerve canal

**Figure 3:**
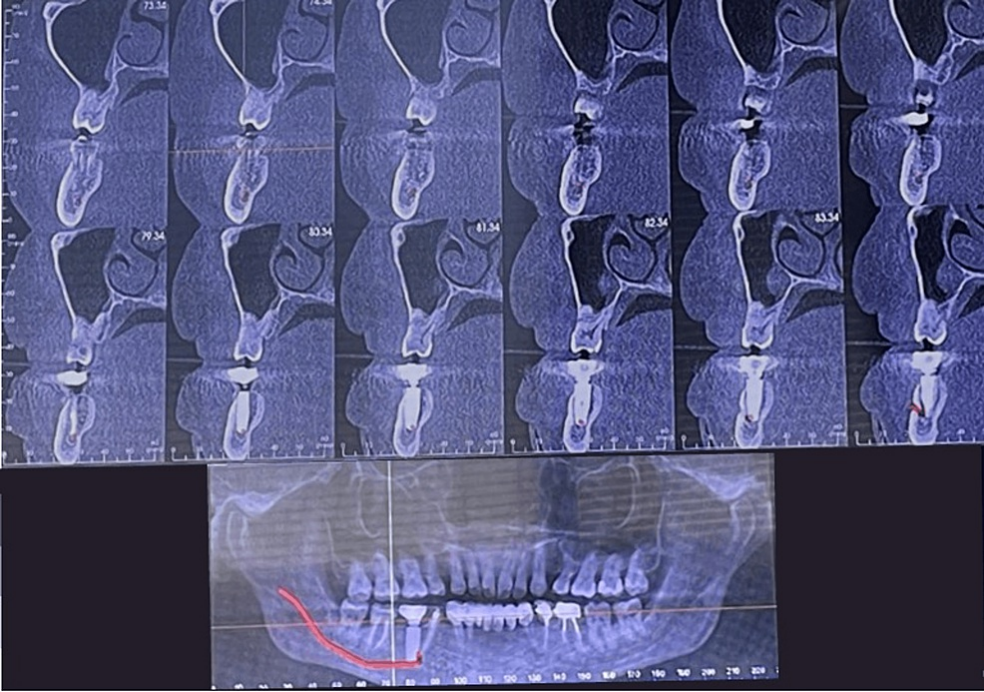
Cone beam computed tomography image showing implant apex impinging on the inferior alveolar nerve canal

To better define the anatomic position of the mandibular canal and the relationship of the canal with the implant apex, the radiological examination was done with an orthopantanogram (OPG) and confirmed with a cone beam computed tomography (CBCT), which revealed that an endosseous implant was placed in relation to 46 and that the implant apex was resting on the inferior alveolar nerve canal (Figures 2, 3).

Therapeutic intervention

**Figure 4:**
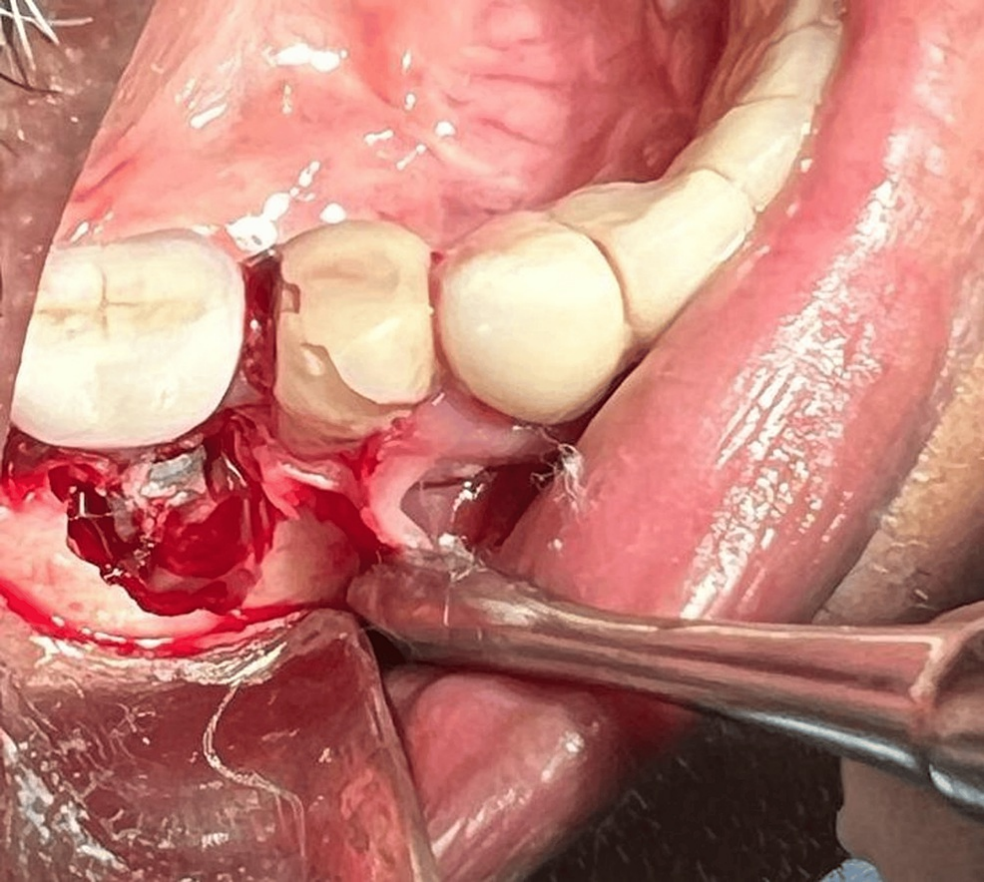
Intra-operative photograph showing the implant and circumferential bone loss in relation to 46 after elevation of the periodontal flaps

**Figure 5:**
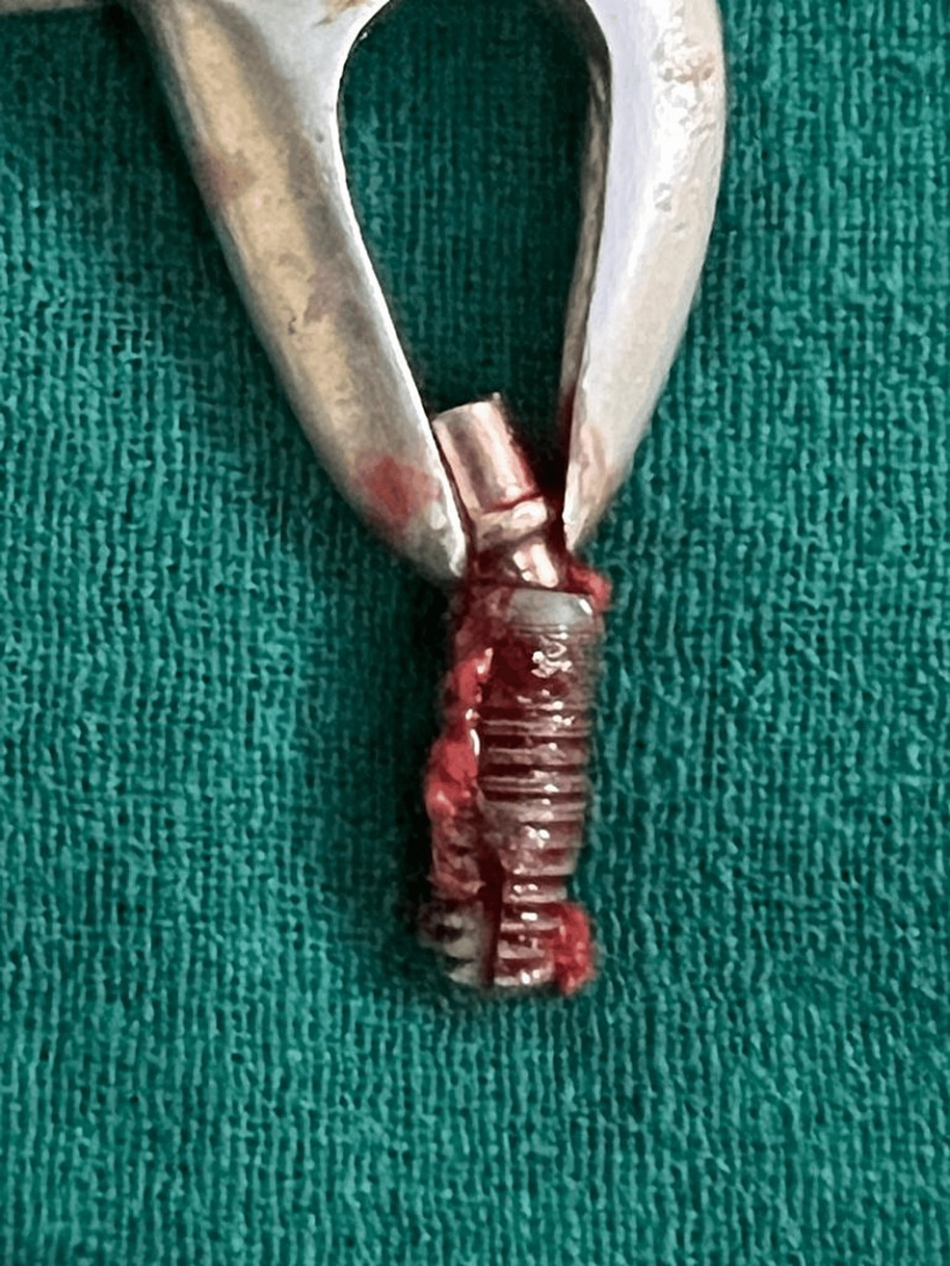
Surgically retrieved implant

**Figure 6:**
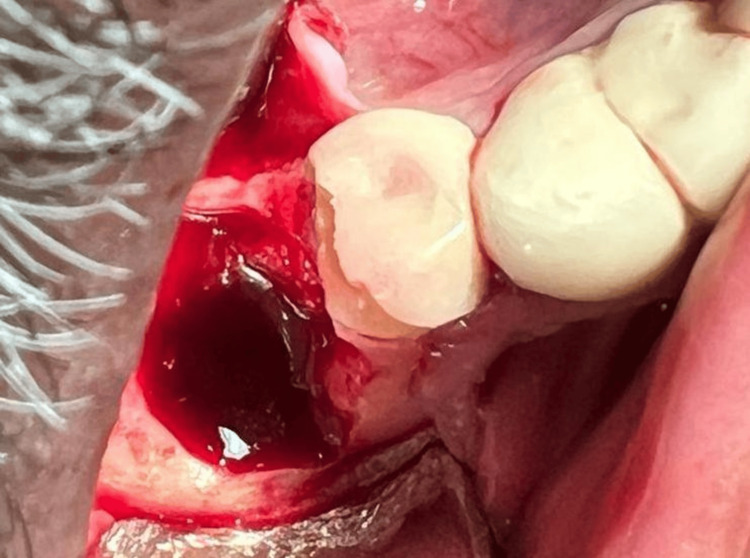
Extracted socket after implant retrieval

**Figure 7:**
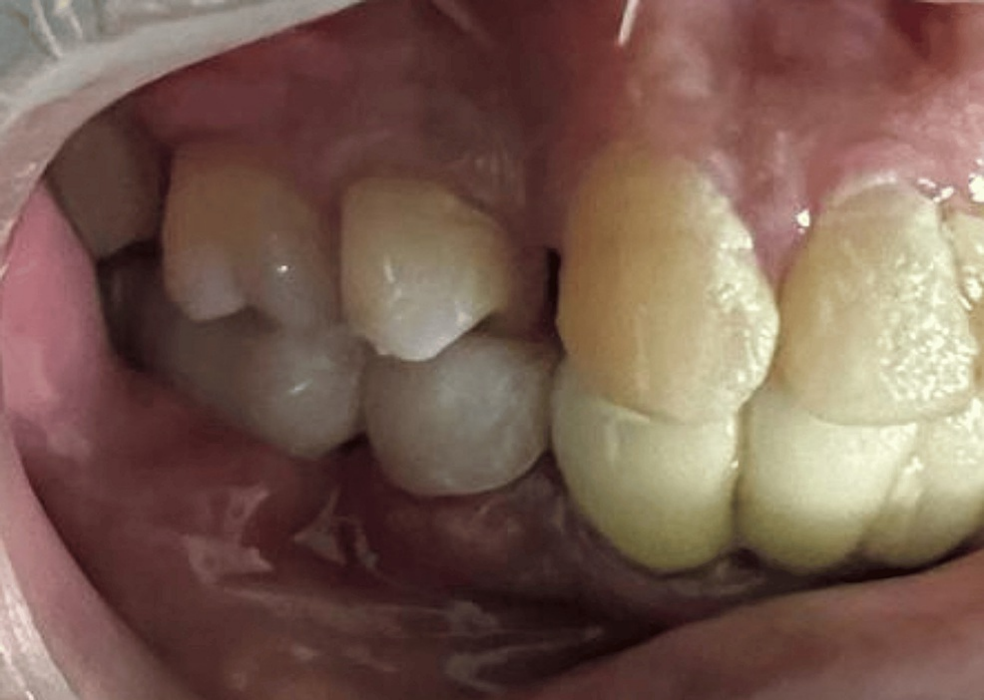
Postoperative photograph showing provisional three-unit fixed partial denture in relation to 45, 46, and 47 and complete re-establishment of neurosensory function

Based on the clinical presentation and radiological examination, a provisional diagnosis of the condition of neurosensory disturbance due to inferior alveolar nerve injury was made. The clinical scenario was completely explained to the patient, and informed consent was obtained. His physician's consent was also obtained owing to his past medical history. Routine hematological investigations were performed, and the values were within the normal range. Under local anesthesia, the prosthetic component was first removed, the buccal and lingual flaps were elevated, and the implant was extracted with extraction forceps. The granulation tissue was completely debrided, resective osseous surgery was performed to create a positive bony architecture, and the flaps were approximated with sutures. While the implant was being removed, the patient expressed a sensation similar to an electric shock, suggesting there may be a connection within the neural network. After a week, the sutures were removed, and in about the fourth week, the edentulous site was restored with a three-unit fixed partial denture (Figures 4-7). 

Postoperative neurosensory evaluation

The objective assessment revealed diminished mechanical sensory abilities such as light touch, differentiation between sharp and blunt stimuli, ability to discern moving points, and ability to distinguish between two points. The patient initially reported a decrease in subjective function in the neuropathic region of the right inferior alveolar nerve, scoring nine on a scale of 1 to 10.

**Figure 8:**
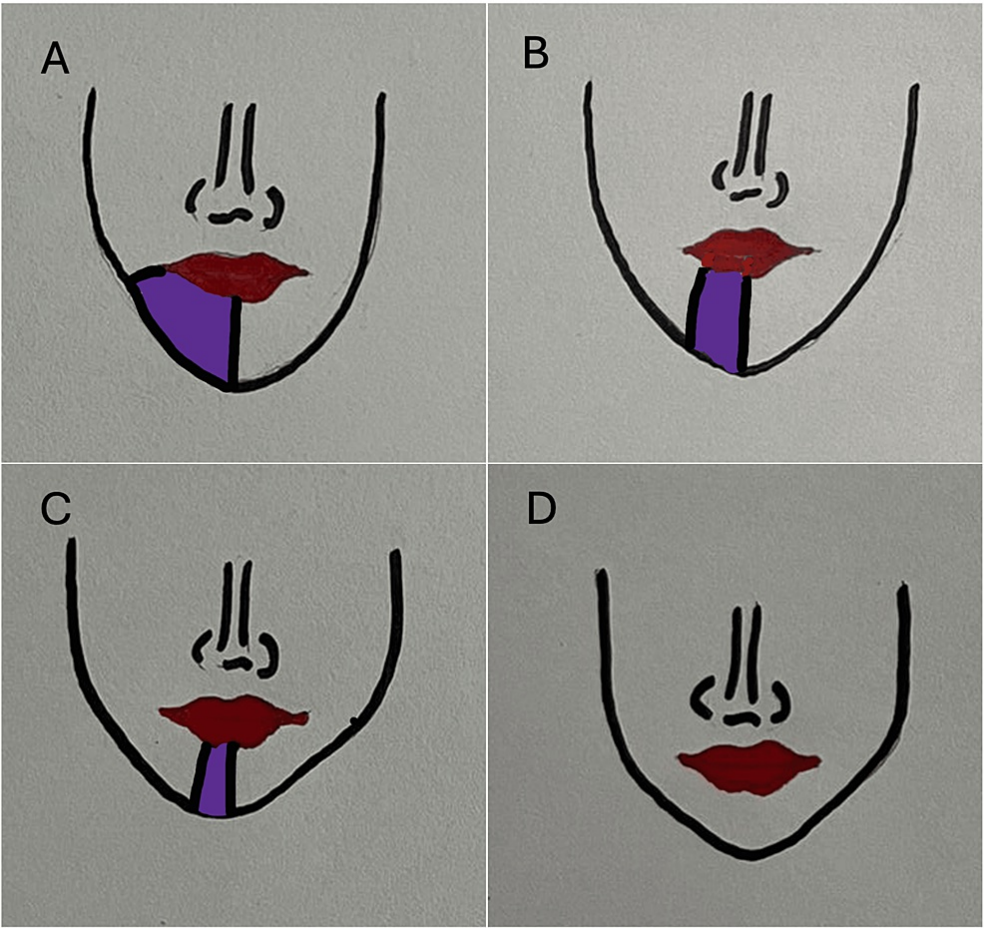
Diagrammatic representation of neurosensory re-establishment as experienced by the patient over the six weeks period A - paraesthesia 100% on day one (preoperative); B - 50% neurosensory recovery at week three; C - 70% recovery at week four; D - 100 % recovered at week six (complete recovery) Note: The colored area represents neurosensory deficit

During the follow-up appointment, three weeks after the surgical retrieval, the area affected with neurosensory disturbance had reduced to approximately half of the neuropathy area. Four weeks after the injury, the patient showed significant improvement in sensation. However, 30% of the affected area still showed a persistent neuro-sensory deficit and exhibited some sensitivity to cold and mechanical stimuli. Nevertheless, by the end of six weeks, her neurosensory deficit has completely resolved (Figure 8, Table 1). In addition, the salivary drooling from the corner of the mouth gradually reduced, and the patient returned to normalcy by this period. The occasional burning sensation and the pain had also completely resolved, as assessed by the Visual Analogue Scale. However, he was monitored for three months to ensure complete recovery.

**Table 1:** Numbness subjective test elicited from the patient

Neurosensory evaluation (numbness) - at various periods	Neurosensory loss in %
Preoperative	100
Day 1	100
Week 1	90
Week 2	70
Week 3	50
Week 4	30
Week 5	10
Week 6	0 - Complete recovery
Week 12	0 - No abnormality detected

## Discussion

Neurosensory injuries can occur at various stages, including administering local anesthetic, flap elevation, osteotomy site preparation, and implant placement. The ensuing morbidity and psychological distress highlight the importance of understanding the impact of current surgical interventions. However, there is still a dearth of guidelines for the surgical management of inferior alveolar nerve injuries associated with dental implants [10].

Several studies have highlighted the various surgical procedures warranted for dental implant-related nerve injuries. While CBCT is used to accurately locate the inferior alveolar canal, magnetic resonance imaging (MRI) is used to visualize the lingual and the inferior alveolar nerve. Nevertheless, surgical exploration is the best method to provide an optimum view of the inferior alveolar nerve injury. This technique determines whether the implant has just contacted the nerve or any bony fragments impinge on it. If an inferior alveolar nerve injury is anticipated during or after the placement of the implant, it is warranted that the implant is removed from the site within the first 36 hours. During the retrieval procedure, care should be taken not to push any bone graft material, if present, onto the injured nerve [11]. Certain complications may arise due to an inferior alveolar nerve injury, and thus, prompt attention is needed. Sometimes, a disorganized axonal regeneration can occur, leading to the formation of a neuroma, which may, in turn, lead to dysesthesia. In such cases, a partial transection of the nerve is to be performed [10].

Reports have suggested that sensory loss due to nerve injury can be relatively improved easily if the implant is removed immediately after the occurrence of sensory loss or any neurosensory disturbance. It was also suggested that the re-establishment or regaining of the sensory loss is directly proportional to the time elapsed since the onset of sensory loss [12]. However, in the present case, although two years have elapsed from implant placement, there was complete recovery within six weeks of implant removal, owing to the fact that the implant apex was merely brushing the surface of the mandibular canal without actually impinging or damaging it. There are also reports that, in cases with long-term sensory loss, the body of the implant is often osseointegrated into the bone, causing difficulty in surgical removal and posing a diminished possibility of symptomatic improvement [11]. However, in the current case, there was a peri-implant bone defect leading to circumferential bone loss around the implant, causing an increase in the probing depth of about 5 mm, possibly diagnosed as peri-implantitis. Thus, the loss of attachment constituted by the bone loss has led to uncomplicated implant retrieval.

Another discomfort in this case was the drooling of saliva, which was also completely resolved in about six weeks, which has also been previously reported [13]. The intermittent pain that the patient experienced was due to the peri-implant bone defect and gingival inflammation, which accumulated sub-gingival plaque that eventually led to dull, intermittent pain. The surgical periodontal flap surgery with complete debridement of supragingival and sub-gingival deposits and the granulation tissue warded off the pain completely in two days following the procedure. This was in accordance with previous reports [14]. The burning sensation, reported as 6 out of 10 on the Visual Analogue Scale, gradually reduced to 0 out of 10 in about two weeks from the surgical management. A previous study has reported a residual burning sensation which measured as one out of 10 on a visual analogue scale [15].

Several studies that have opted for extraction of dental implants after inferior alveolar nerve injury demonstrated that patients recovered with complete neurosensory re-establishment [16, 17]. The highest success rates were observed with early removal, within one and a half days after implant placement [11, 16]. Some studies noted that removing implants after one and a half days did not lead to successful or rather complete recovery [18]. However, some studies on delayed implant extraction have also reported successful sensory recovery. A previous study with an implant retrieved two months after placement resulted in complete recovery [17]. Thus, understanding the incidence rate of this condition and its clinical progression post-surgery can enhance treatment success, improve patient cooperation, and increase patient satisfaction. 

To summarise, the probable causes of inferior alveolar nerve injury during implant placement include mechanical trauma from injection needles, the implant or the implant drill, debris formed during osteotomy site preparation, hematoma formation in the inferior alveolar nerve canal, improper incision and injudicious instrumentation, especially during soft tissue retraction. These factors may damage the nerve due to pressure, encroachment, or laceration. In addition, an intrusion of the implant or the drill into the canal may cause compression of the nerve, resulting in secondary ischemia and subsequent nerve degeneration.

In order to avoid these complications, it is prudent to have a fundamental understanding of the jaw anatomy, supported by a comprehensive clinical and radiological examination. It is noteworthy that clinicians may undertake a neurosensory examination of the nerve function before the inception of the procedure to rule out any prior neurosensory deficit. Appropriate measurement of the bone height coronal to the mandibular canal is one of the best ways to avoid such injury. In addition, the use of CBCT-based surgical guides may help prevent injury. Furthermore, Heller et al. have advocated the practice of local anesthetic infiltration technique over nerve blocks with a notion that, under infiltration, there is no complete lack of sensation, and thus, if the drill approaches close to the canal, the patient will experience pain, an absolute indication to stop further drilling [19]. By and large, a thorough understanding of the anatomy, surgical procedure, and implant system, along with proper diagnosis and treatment planning, will ultimately abate the occurrence rate of inferior alveolar nerve injury. However, if this occurs, prompt management is the key to augment the chances of complete recovery.

## Conclusions

Nerve injuries during implant procedures, at times, are unpredictable and can occur despite meticulous presurgical planning aimed at avoiding critical structures. It is essential to recognize the inherent risk of nerve damage, implement appropriate preventive measures, and thoroughly inform patients about these risks to mitigate liability. This clinical report underscores the fact that even with comprehensive treatment planning, exceptional expertise, and advanced technology, complications such as nerve injury can still arise. Nonetheless, the emphasis remains on prevention, as avoiding nerve injury is far superior to managing its consequences.
